# A retrospective comparative study of progression-free survival and overall survival between metachronous and synchronous metastatic renal cell carcinoma in intermediate- or poor-risk patients treated with VEGF-targeted therapy

**DOI:** 10.18632/oncotarget.20674

**Published:** 2017-09-06

**Authors:** Sung Han Kim, Yoon Seok Suh, Dong-Eun Lee, Boram Park, Jungnam Joo, Jae Young Joung, Ho Kyung Seo, Kang Hyun Lee, Jinsoo Chung

**Affiliations:** ^1^ Department of Urology, Center for Prostate Cancer, Research Institute and Hospital of National Cancer Center, Goyang, Korea; ^2^ Biometric Research Branch, Division of Cancer Epidemiology and Prevention, Research Institute and Hospital of National Cancer Center, Goyang, Korea

**Keywords:** renal cell carcinoma, metastasis, synchronous, metachronous, prognosis

## Abstract

**Introduction:**

The aim of this study was to compare progression-free survival (PFS) and overall survival (OS) between metachronous and synchronous metastatic renal cell carcinomas treated with VEGF-targeted therapy.

**Methods:**

Between 2005 and 2014, 93 (78.8%) intermediate- and 25 (21.2%) poor-Heng-risk patients, including 32 (27.1%) patients with metachronous and 86 (72.9%) patients with synchronous renal cell carcinoma, were enrolled retrospectively. PFS and OS values were compared according to the number of risk factors and treatment-free interval using the Kaplan-Meier method and log-rank test. The prognostic risk factors were also evaluated using a Cox proportional hazard model, with a p-value < 0.05 indicating statistical significance.

**Results:**

During a median 5.0-month treatment and 59.3-month follow-up, analysis of the PFS/OS of SM (5.2/9.6 months) and MM (9.6/20.1 months) yielded a significant difference in OS (p = 0.010). However, there was no significant difference when Heng risk groups and treatment-free interval were considered (p > 0.05). There was a significant difference in PFS (hazard ratio: 1.81) and OS (hazard ratio: 2.19) with increasing number of Heng risk factors among patients with synchronous renal cell carcinoma and a treatment-free interval <1 year. Metastatic type, anemia, and neutrophilia were significant predictive factors for OS in multivariable analysis (p < 0.05).

**Conclusion:**

The metastatic type of renal cell carcinoma (synchronous or metachronous) significantly affects survival; metachronous type is associated with more favorable outcomes than synchronous type. However, after stratification according to Heng risk factors and treatment-free interval, the differences in survival between metachronous and synchronous type were insignificant.

## INTRODUCTION

Approximately 20–30% of all metastatic renal cell carcinomas (mRCCs) are synchronous mRCCs (SMs), a newly diagnosed metastatic entity. A further 20% of mRCCs are metachronous mRCCs (MMs), a recurring or progressed localized RCC that has metastasized after curative surgical therapy [1, 2]. In past decades, the standard therapeutic approach for mRCC was systemic therapy using cytokines, and a shift toward targeted therapy (TT) has occurred over the last recent decade owing to an increased understanding of the pathogenesis of RCC provided by next-generation sequencing [3]. In contrast to cytokine therapy, recently developed TT focuses more on the treatment of a highly vascularized and immune-related tumor microenvironment [3, 4]. However, the different pathophysiologic microenvironments and immune-related conditions of different metastatic types produce various therapeutic responses to TT in the clinical context of mRCC [4]. This has resulted in a 0–20% 5-year survival rate, which is considered unacceptable by most clinicians [3, 5, 6].

The response of SM and MM to TT is variable owing to the pleomorphic nature of RCC and diverse tumor burden levels associated with the disease [7, 8]. MM probably involves fewer oncogenic events than SM, especially in primary tumors. The dormant tumor cells in MM experience oncogenic activity, which stimulates them to form and grow at the site of metastases at a certain point in time following nephrectomy [4, 9]. Previous immunohistochemical tissue studies have demonstrated that SM has distinct phenotypes with different oncogenic events, resulting in worse prognoses than MM [4, 10]. These unpredictable and diverse characteristics of mRCC, especially in different metastatic types, are difficult to predict from the primary tumor alone, but easier to predict based on combined data from metastatic tumors. Therefore, understanding the prognostic differences between SM and MM is important for developing strategies for treating mRCC in the TT era [11, 12].

The general consensus is that the prognosis of MM is better than that of SM. However, no objective data regarding this issue have been published, except for some case reports, retrospective studies of specific organ metastases, and genetic analyses of specific RCC histological types [4, 13-16]. Clinicians require relevant prognostic data in order to understand the prognoses of patients with MM or SM and to treat them effectively with TT.

With the above in mind, this study aimed to retrospectively analyze the progression-free survival (PFS) provided by first-line VEGF-TT and the overall survival (OS) of MM and SM patients according to Heng risk groups [10]. The two groups were divided into sub-groups based on treatment-free intervals (TFIs) and analyzed further. Finally, we assessed the predictors of OS and compared the results between MM and SM.

## RESULTS

The median treatment period was 5.0 months (1.0–62.0) and the median follow-up period was 59.3-months (4.8–61.3). Only 20 (17.0%) patients survived this period. The median PFS and OS (equal to cancer-specific survival) were 5.6 (1.0–61.3) and 12.8 (1.0–87.2) months, respectively (Supplementary Table 1). The rate of nephrectomy and metastasectomy were 45.8% and 19.5%, respectively. Metastatic lesions were most prevalent in the lungs (72%), followed by the lymph nodes (42.4%), bone (35.6%), liver (21.2%), and brain (12.7%) (Supplementary Table 1). MM was associated with significantly higher rates of nephrectomy, TFI ≥1 year, clinical T3-4 and N1 stages, and G3-4 nuclear grades (p < 0.05, Table 1).

**Table 1:** Comparison of baseline characteristics between metachronous (N=86) and synchronous (N=32) mRCC groups with exclude non-clear cell patients

	Metachronous (N= 86)	Synchronous (N= 32)	P-value
Age (years)	58.73±10.63	57.31±11.39	0.528^a^
Gender (male/female)	69/17 (80.2/19.8)	25/7 (78.1/21.9)	0.800^b^
Nephrectomy	23 (27.4)	31 (100)	<0.001^c^
Metastatectomy	19 (22.1)	4 (12.5)	0.303 ^c^
TFI, ≥1yr/ <1yr	5/81 (5.8/94.2)	22/10 (68.8/31.3)	<0.001^b^
Anemia	59 (68.6)	27 (84.4)	0.088^b^
Hypercalcemia	11 (13.3)	4 (12.5)	1 ^c^
Neutrophilia	13 (15.5)	9 (28.1)	0.120^b^
Elevated LDH	20 (30.8)	6 (26.1)	0.793^b^
KPS > 80 /≤ 80	83/3 (96.5/3.5)	32/0 (100/0)	0.559^c^
Thrombocytosis	11 (12.8)	3 (9.4)	0.756 ^b^
Creatinine	1.2±0.4	1.3±0.5	0.210
eGFR	72.6±21.0	70.7±22.2	0.667
Metastatic lesion			
Lung	58 (67.4)	27 (84.4)	0.105 ^c^
Liver	17 (19.8)	8 (25.0)	0.614 ^c^
Lymph node	38 (44.2)	12 (37.5)	0.538 ^c^
Bone	31 (36.0)	11 (34.4)	1.000 ^c^
Brain	10 (11.6)	5 (15.6)	0.547 ^c^
Heng, Intermediate risk	65 (75.6)	28 (87.5)	0.159 ^b^
Poor risk	21 (24.4)	4 (12.5)	
cT or pT stage, T1-T2/ T3-T4	42/22 (65.6/34.4)	9/15 (37.5/62.5)	0.017^b^
cN or pN stage, N0/ N1/ Nx	30/19/15 (46.9/29.7/23.4)	0/11/0 (0/100/0)	<0.001^b^
Fuhrman nuclear grade,			
G1-G2/G3-G4	22/35 (38.6/61.4)	3/20 (13/87)	0.026 ^b^
Treatment duration (Mos.)	7.44±10.05	11.96±14.69	0.116 ^a^
Follow-up duration (Mos.)	59.3 (4.8-60.1)	61.3 (9.4-64.3)	0.075 ^d^
Progression-free survival (Mos)	5.2 (1.0-60.4)	9.6 (1.0-62.0)	0.059 ^d^
Overall survival (=Cancer specific survival, Mos.)	9.6 (1.0-62.3)	20.1 (1.5-87.2)	0.010 ^d^
Survival/death	15/71 (17.4/82.6)	5/27 (15.6/84.4)	1^b^

a: Student's t-test, b: Chi-square test, c: Fisher exact test, d: Log-rank test, TFI: Treatment free interval, KPS: Karnofsky performance status, mos: months.

**Figure 1:**
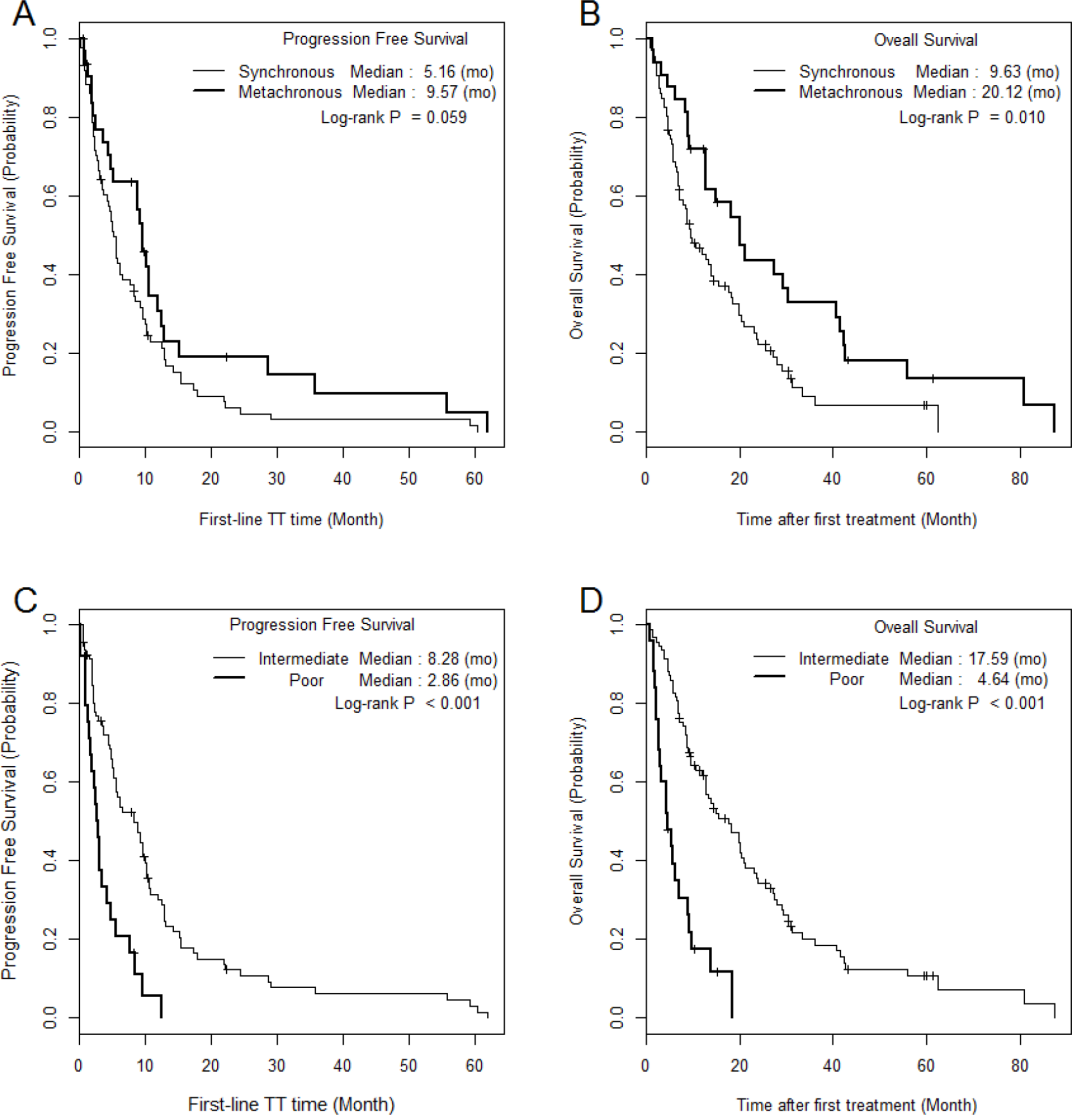
The comparison of Kaplan Meier survival curves according to metastatic type and Heng risk groups

**Figure 2:**
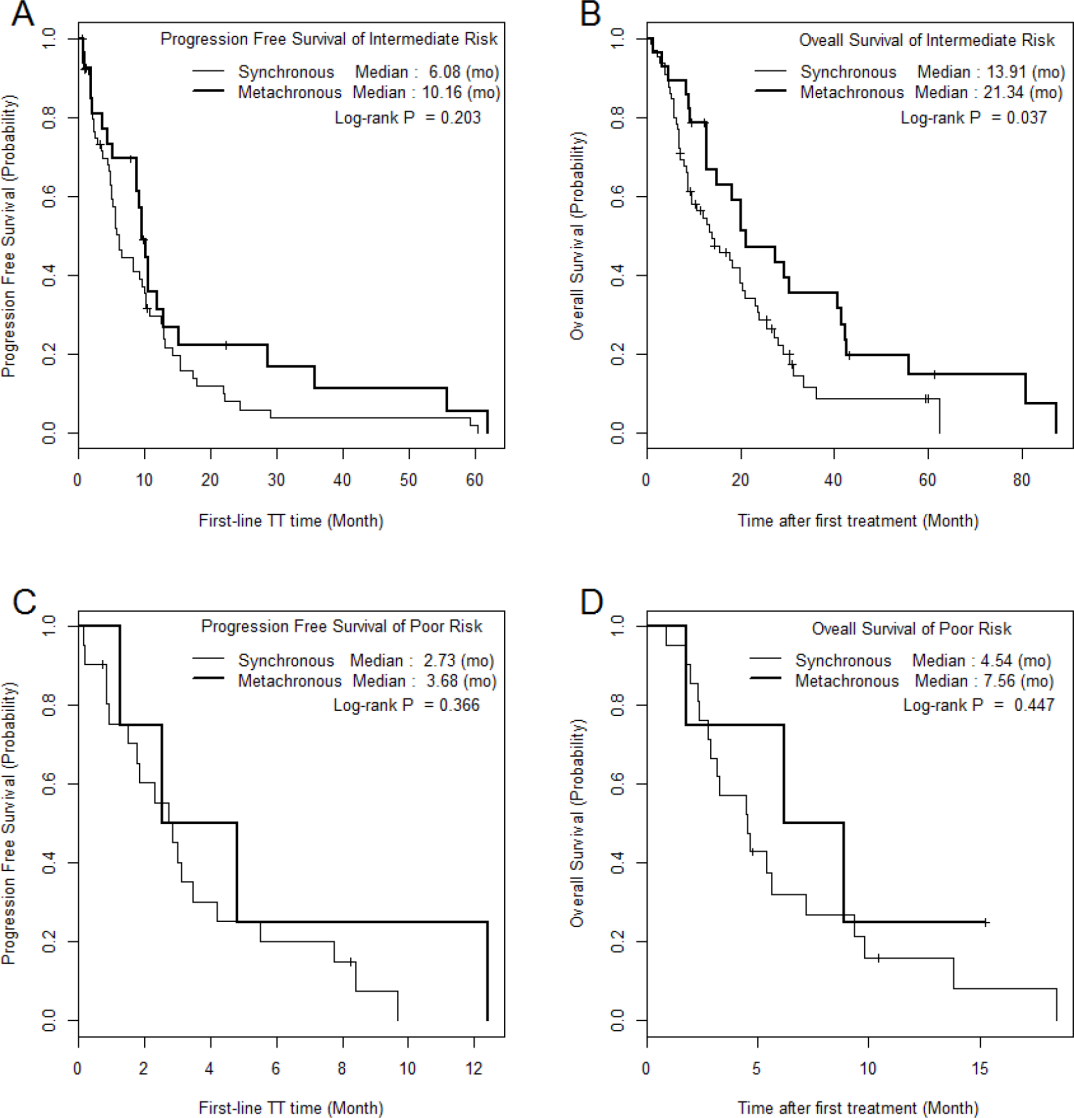
The comparison of Kaplan Meier survival curves according to mRCC group in each Heng risk group

**Figure 3:**
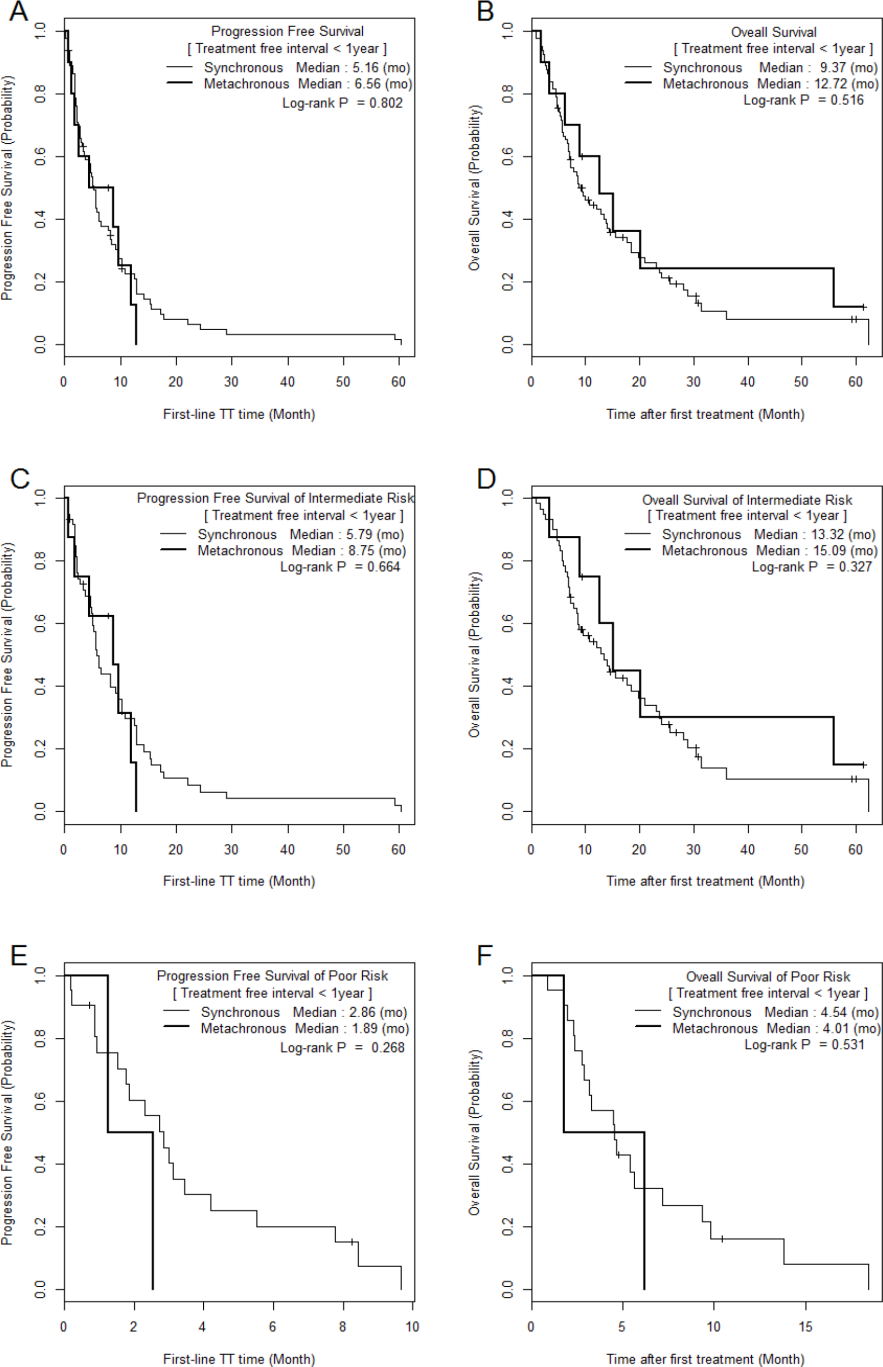
Comparison of Kaplan Meier survival curves between MM and SM groups with treatment-free interval < 1 year

**Figure 4:**
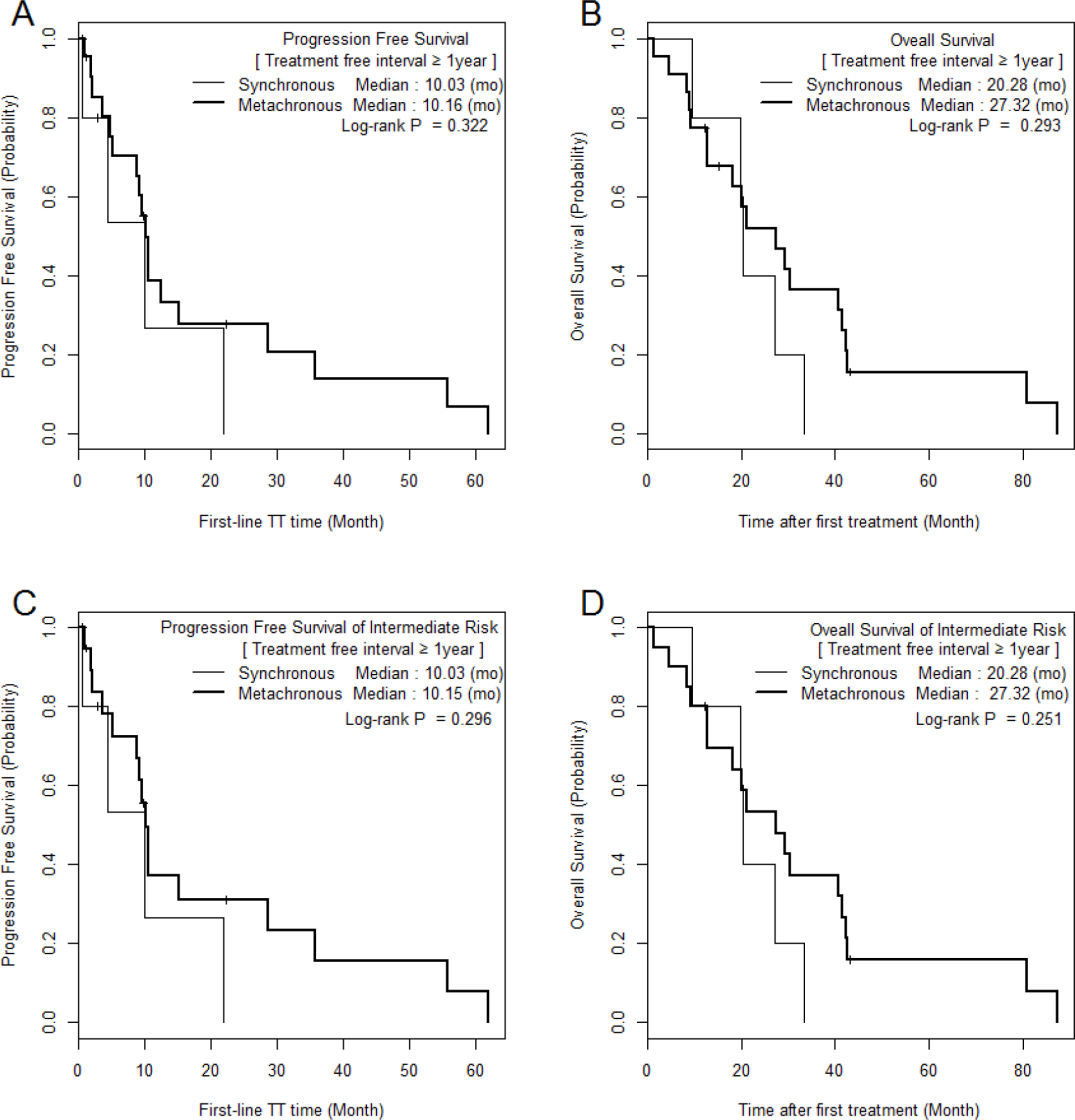
Comparison of Kaplan Meier survival curves between MM and SM groups with treatment-free interval ≥ 1 year

The median PFS and OS of SM/MM patients were 5.2 (1.0–60.4)/9.6 (1.0–62.0) (p = 0.059) and 9.6 (1.0–62.3)/20.1 (1.5–87.2) months (p = 0.010), respectively (Figure 1). The median PFS and OS of the intermediate-risk group (8.3/17.6 months) were significantly higher than those of the poor-risk group (2.9/4.6 months) (p < 0.01). The PFS and OS values of SM/MM patients according to intermediate and poor Heng risk (Figure 2), TFI <1 year (Figure 3), and TFI ≥1 year (Figure 4) were not significantly different (p > 0.05), although we did not compare PFS and OS in SM patients with TFI ≥1 year owing to the small numbers of cases (Figure 4).

The effect of the number of Heng risk factors on PFS and OS was analyzed in SM and MM patients according to the two TFI subgroups. Increasing number of Heng risk factors had a significant effect on PFS (hazard ratio [HR]: 1.81; 95% confidence interval [CI]: 1.37–2.38) and OS (HR: 2.19; CI: 1.63–2.94) in SM patients with TFI <1 year (p < 0.001, Table 2). We did not assess the effect of increasing numbers of risk factors in SM patients with TFI ≥1 year owing to small patient numbers. No significant effect of increasing numbers of Heng risk factors was observed for PFS or OS in MM (p > 0.05, Table 2). A Cox proportional hazards model showed that metastatic type (SM/MM) (HR: 2.72; CI: 1.64–4.51), neutrophilia (HR: 3.21; CI: 1.76–5.87), and anemia (HR: 2.48; CI: 1.49–4.13) were significant predictive factors for OS (p < 0.05, Table 3).

**Table 2:** The hazard ratios of Heng risk factor for PFS and OS in SM/MM patients stratified by TFI

			HR (95% CI)	p-value
Total set	Synchronous	PFS	1.82 (1.38-2.39)	<.001
		OS	2.25 (1.67-3.04)	<.001
	Metachronous	PFS	0.96 (0.52-1.78)	0.999
		OS	1.13 (0.57-2.24)	0.754
TFI < 1 year set	Synchronous	PFS	1.81 (1.37-2.38)	<.001
		OS	2.19 (1.63-2.94)	<.001
	Metachronous	PFS	1.6 (0.58-4.43)	0.385
		OS	2 (0.65-6.19)	0.159
TFI ≥ 1 year set	Synchronous	PFS	N.A.	N.A
		OS	N.A.	N.A
	Metachronous	PFS	0.93 (0.43-2.02)	0.928
		OS	1 (0.42-2.41)	0.905

HR; hazard ratio, CI; confidence interval, TFI; treatment-free interval, PFS; progression-free survival, OS; overall survival; N.A, not available due to small number of patients in SM group with TFI ≥ 1 year set.

**Table 3:** The Cox proportional hazard model of predictive factors of overall survival in metastatic renal cell carcinoma

	Univariable		Multivariable	P-value
	Hazard ratio (95% CI)	P-value	Hazard ratio (95% CI)	
	N = 118 / EVENT= 98		N=116 / EVENT=96	
Metastatic type SM	1.84 (1.15-2.94)	0.011	2.72 (1.64-4.51)	<0.001
Treatment Free interval <1yr	1.88 (1.16-3.06)	0.010		
Anemia	1.87 (1.14-3.06)	0.013	2.48 (1.49-4.13)	0.001
Hypercalcemia	1.20 (0.69-2.09)	0.528		
Neutorphilia	1.64 (0.96-2.8)	0.071	3.21 (1.76-5.87)	<0.001
Elevated LDH	1.20 (0.7-2.04)	0.506		
KPS≤ 80	1.50 (0.37-6.12)	0.574		
Thrombocytopenia	2.09 (1.10-3.99)	0.025		

KPS, Karnofsky performance status.

## DISCUSSION

Few reports have investigated the overall prognoses of metastatic types treated with TT, especially using stratification methods such as prognostic risk models [5, 9, 12]. Variable degrees of tumor burden, unpredictable and diverse outcomes, and the disproportionate number of SM and MM cases treated with TT have so far limited the analysis of long-term survival. Previous mRCC studies have demonstrated that MM is associated with better prognostic outcomes than SM [6-8, 10, 13, 16], but patients were not assessed using survival analysis of stratified risk factors. These previous analyses mostly involved patients with favorable and intermediate risks, resulting in better results for MM than for SM.

In the present study, anemia and neutrophilia were identified by multivariable analysis as predictive factors of overall survival, and these are known prognostic factors [22]. The anemia-induced hypoxic tumor microenvironment might lead to mutations, resulting in increased treatment resistance and increased metastatic potential signals, and tumor-associated neutrophilia neutrophils might be crucial for remodeling the tumor microenvironment, resulting in worse oncological features and cancer mortality [23]. We also collected some interesting data concerning survival between SM and MM according to different prognostic risk models and TFI [22, 24]. A significant difference in OS between MM and SM was observed, but no significant difference was observed when we stratified the data by TFI and risk factors (Figures 3 and 4). This analysis showed that in the intermediate-risk group, SM was associated with a significant improvement in OS compared to that in the MM (21.3 vs. 13.9 months, p = 0.037; Figure 2B). The Cox proportional hazards model assessing the prognostic effect of the number of Heng risk factors on survival also showed significance only in SMs with TFI <1 year (p < 0.001), whereas MM was not significantly affected by an increasing number of Heng risk factors (p > 0.05, Table 2). The results of this study, when combined with those from our previous reports [12, 17], suggest that different therapeutic strategies may be required for MM and SM.

Some questions regarding the results of this study should be answered. First, what underlies the differences in survival between SM and MM despite the lack of significant differences in prognostic risk factors and TFI subgroups? The inconsistent relationship between SM/MM and OS may be explained by differences in the baseline characteristics of patients with the different metastatic types. There were more than twice as many patients with poor-risk in the SM group (24.4% vs. 12.5%, p = 0.159) (Table 1), and an insufficient number of patients with intermediate risk may have influenced the difference in survival between patients with SM and MM.

The second issue to discuss is the fact that TFI was not a significant prognostic risk factor in this study (not selected through backward elimination, Table 3), whereas a previous large-scale IMDC study suggested otherwise [10]. In this previous study, 33.2% of patients had previously received immunotherapy and 82.5% had undergone nephrectomy, whereas in the present study, only 48.1% had undergone nephrectomy and none had undergone immunotherapy. Another reason was that during TFI analysis, intermediate and poor-risk patients were re-classified according to their TFI in order to homogenize the SM and MM groups. Because TFI indirectly refers to disease aggressiveness or progression rate, which lead in turn to unfavorable OS in mRCC [25], there were insignificant differences in survival between the SM and MM groups when controlling for growth rate and tumor activity. However, SM and MM patients with a TFI <1 year had worse PFS/OS than those with a TFI ≥1 year (Figures 2 and 3). Furthermore, an increasing number of Heng risk factors significantly and negatively affected OS only in SM patients with TFI <1 year (p < 0.001, Table 2). In those with TFI ≥1 year, intermediate-risk mRCC was associated with a similar median OS in MM and SM, suggesting that the TT strategy may be similar in those with slowly progressing mRCC regardless of metastatic type. Meanwhile, a different strategy may be applied in cases of MM and SM in patients in whom mRCC is progressing faster (TFI <1 year). Considering the results of the present study and those of previous tissue microarray and clinicopathological studies [12, 17, 21, 24], TT strategies should be developed with consideration of the patient’s metastatic type and TFI.

The small number of poor-risk patients precluded the analysis of survival in patients with TFI ≥1 year (Figure 3) because most patients with poor risk were in the SM with TFI <1 year group (24.4% vs. MM 12.5%), and no prognostic differences were observed between SM and MM. Those with poor risk were mostly in a bad general condition, with anemia and neutrophilia, and had fast-developing disease. We believe that the TT strategy for poor-risk patients should focus on improving the patient’s general condition while taking into account the tolerability of target agents, and should attempt to suppress the fast-growing tumor. Future, large-scale, translational analyses, including genetic analysis and novel metabolic assessment techniques, will be needed.

Another difference between SM and MM is the presence or absence of primary tumors following nephrectomy. In the present study, in all patients with MM who had undergone nephrectomy, MM was detected early during a regular follow-up after nephrectomy, and the disease had a lower tumor burden than SM, which was associated with the tumor burden of the primary tumor and a relatively large tumor burden in the metastatic tumor. Our additional analysis of nephrectomy in patients with SM and MM showed that the SM (20.0 months) and MM (20.1 months) groups were associated with similar median values of OS (data not shown). The degree of tumor burden, including that of the primary tumor, was an important prognostic factor. This implies that mRCC treated by TT once the primary tumor has been removed is associated with similar prognoses regardless of metastatic type. Furthermore, cytoreductive nephrectomy seems to be a more important prognostic factor for mRCC [26, 27]. The decreased tumor burden that occurred with the disappearance of the primary tumor lesion provided better prognostic outcomes with systemic TT. However, good performance status and a primary tumor accounting for >75% of the overall tumor burden without any metastatic lesions in the central nervous system and liver did not yield a better prognostic outcome for every mRCC patient after cytoreductive nephrectomy, which resulted in a low rate of nephrectomy in the targeted era [28, 29].

The current study was limited in a few key ways, mainly by the retrospective study design and small number of enrolled patients in each mRCC group, especially in the SM and poor-risk groups. The intermediate-risk group should ideally be investigated according to pathophysiologic characteristics between and within primary tumors after nephrectomy and metastatic tumors after metastasectomy, especially in the case of MM. Metabolic tumor activity, nephrectomy, overall tumor burden including that of metastatic organs, gene sequencing analysis, and the effect of different medical targeted agents were not considered in our analysis.

In conclusion, the metastatic type (SM or MM) of mRCC appears to be a significant prognostic risk factor in TT-treated mRCC patients. A non-significant favorable survival trend was observed in patients with MM, especially in intermediate-risk patients. However, after stratifying patients according to TFI, the Heng intermediate-risk group was not associated with any significant differences between SM and MM patients with TFI <1 year. This may suggest that TFI, or the rate of progression, has a greater influence on survival than metastatic type. However, based on the findings of this study, we suggest that both metastatic type and TFI should be considered when clinicians develop TT strategies for their patients.

## MATERIALS AND METHODS

### Ethics statement

The institutional review board of the National Cancer Center (IRB No. NCC2016-0263) approved this retrospective study, and waived the requirement for written informed consent. All patient data were anonymized and de-identified prior to analysis. All study protocols were performed in accordance with the ethical tenets of the Declaration of Helsinki.

### Patient characteristics

From 2005 to 2014, we retrospectively reviewed 93 (78.8%) intermediate (1–2 Heng risk factors) and 25 (21.2%) poor (≥3 Heng risk factors) Heng risk patients, including 32 MM patients (27.1%) and 86 SM patients (72.9%). The exclusion criteria were non-clear cell RCC and favorable risk, because the SM group consisted only of intermediate-risk and poor-risk patients. The patients’ baseline information, including the metastatic lesions, rate of nephrectomy, and metastasectomy, are described in Supplementary Table 1. The choice of systemic VEGF-target agents was made at the discretion of the treating urologist (JC) according to pathological findings and the patient’s national insurance coverage, as cited in a previous study [17].

The International Metastatic Renal Cell Carcinoma Database Consortium risk (IMDC risk, also known as the Heng risk) criteria [10] were used for prognostic risk stratification to predict the response to systemic therapy among patients with mRCC. Prognostic factors from the Heng risk criteria included hemoglobin level < lower normal limit, Karnofsky performance score <80%, thrombocytosis > upper normal limit (ULN), neutrophilia > ULN, hypercalcemia > ULN, and <1 year from diagnosis to treatment (or treatment-free interval, TFI). Fuhrman nuclear grade [18] and TNM stages from the latest International Union Against Cancer classification (2009) [19, 20] were used for pathological RCC evaluation. We used the RECIST criteria v1.1 to evaluate treatment response [21]. All intermediate- and poor-risk mRCC patients underwent a complete evaluation after every two cycles of TT. The follow-up protocol, including all laboratory and imaging evaluations, were based on cycles of target agents described previously [17]. Treatment continued until disease progression was identified using RECIST criteria. Disease progression was defined as at least a 20% increase in the sum of the longest diameter of target lesions in CT imaging.

### Statistical analysis

Descriptions of the baseline characteristics are presented as a median with range (min-max) for continuous variables and a frequency with percentage for categorical variables. The comparison between SM and MM was performed using Student’s t-test for continuous variables and Pearson’s Chi-square test or Fisher’s exact test as appropriate. For PFS and OS, the survival curves were estimated using Kaplan-Meier method and differences between groups were tested using the log-rank test. The survival curves between SM and MM were also compared in subgroups of patients stratified by Heng risk groups (intermediate or poor) and TFI (< 1yr or ≥1yr). The univariable Cox proportional hazard model was performed to estimate the hazard ratio for each 1-unit increase in the Heng risk factors and other risk factors. Variables with a p-value < 0.2 from the univariable model were selected, and the multivariable Cox proportional hazard model was fitted using a backward variable selection method with an elimination criterion of p-value > 0.05. P-values less than 0.05 were considered statistically significant. Statistical analyses were performed using SAS (version 9.3; SAS Institute Inc., Cary, NC, USA) and R-project for statistical computing (version 3.3.2).

## SUPPLEMENTARY MATERIALS TABLE



## Abbreviations

PFS: progression-free survival
OS: overall survival
mRCC: metastatic renal cell carcinomas
SMs: synchronous mRCCs
MMs: metachronous mRCCs
TT: targeted therapy
TFIs: treatment-free intervals
HR: hazard ratio
CI: confidence interval
IMDC: International Metastatic Renal Cell Carcinoma Database Consortium
ULN: upper normal limit

## References

[1] Eggener SE, Yossepowitch O, Pettus JA, Snyder ME, Motzer RJ, Russo P (2006). Renal cell carcinoma recurrence after nephrectomy for localized disease: predicting survival from time of recurrence. J Clin Oncol.

[2] Motzer RJ (2011). New perspectives on the treatment of metastatic renal cell carcinoma: an introduction and historical overview. Oncologist.

[3] Molina AM, Nanus DM (2016). Recent advances in the management of renal cell carcinoma. F1000Res.

[4] Kammerer-Jacquet SF, Brunot A, Pladys A, Bouzille G, Dagher J, Medane S, Peyronnet B, Mathieu R, Verhoest G, Bensalah K, Edeline J, Laguerre B, Lespagnol A (2016). Synchronous Metastatic Clear-Cell Renal Cell Carcinoma: A Distinct Morphologic, Immunohistochemical, and Molecular Phenotype. Clin Genitourin Cancer.

[5] Jonasch E, Gao J, Rathmell WK (2014). Renal cell carcinoma. BMJ.

[6] Motzer RJ, Escudier B, Choueiri TK (2016). Treatment of Advanced Renal-Cell Carcinoma. N Engl J Med.

[7] Bedke J, Gauler T, Grunwald V, Hegele A, Herrmann E, Hinz S, Janssen J, Schmitz S, Schostak M, Tesch H, Zastrow S, Miller K (2016). Systemic therapy in metastatic renal cell carcinoma. World J Urol.

[8] Dabestani S, Marconi L, Bex A (2016). Metastasis therapies for renal cancer. Curr Opin Urol.

[9] Wuttig D, Baier B, Fuessel S, Meinhardt M, Herr A, Hoefling C, Toma M, Grimm MO, Meye A, Rolle A, Wirth MP (2009). Gene signatures of pulmonary metastases of renal cell carcinoma reflect the disease-free interval and the number of metastases per patient. Int J Cancer.

[10] Heng DY, Xie W, Regan MM, Warren MA, Golshayan AR, Sahi C, Eigl BJ, Ruether JD, Cheng T, North S, Venner P, Knox JJ, Chi KN (2009). Prognostic factors for overall survival in patients with metastatic renal cell carcinoma treated with vascular endothelial growth factor-targeted agents: results from a large, multicenter study. J Clin Oncol.

[11] Callea M, Albiges L, Gupta M, Cheng SC, Genega EM, Fay AP, Song J, Carvo I, Bhatt RS, Atkins MB, Hodi FS, Choueiri TK, McDermott DF (2015). Differential Expression of PD-L1 between Primary and Metastatic Sites in Clear-Cell Renal Cell Carcinoma. Cancer Immunol Res.

[12] Kim SH, Park EY, Park B, Joo J, Joung JY, Seo HK, Lee KH, Chung J (2016). The Correlation of Tissue-Based Biomarkers in Primary and Metastatic Renal Cell Carcinoma Lesions: A Tissue Microarray Study. Korean Journal of Urological Oncology.

[13] Decoene J, Ameye F, Lerut E, Oyen R, Van Poppel H, Joniau S (2011). Renal cell carcinoma with synchronous metastasis to the calcaneus and metachronous metastases to the ovary and gallbladder. Case Rep Med.

[14] Ku JH, Moon KC, Kwak C, Kim HH (2009). Metachronous metastatic potential of small renal cell carcinoma: dependence on tumor size. Urology.

[15] Peters I, Hora M, Herrmann TR, von Klot C, Wegener G, Stransky P, Hes O, Kuczyk MA, Merseburger AS (2013). Incidence of synchronous and metachronous adrenal metastases following tumor nephrectomy in renal cell cancer patients: a retrospective bi-center analysis. Springerplus.

[16] Gutenberg A, Nischwitz MD, Gunawan B, Enders C, Jung K, Bergmann M, Feiden W, Egensperger R, Keyvani K, Stolke D, Sure U, Schroeder HW, Warzok R (2014). Predictive chromosomal clusters of synchronous and metachronous brain metastases in clear cell renal cell carcinoma. Cancer Genet.

[17] Kim SH, Park WS, Joung JY, Seo HK, Lee KH, Chung J (2016). Systemic Treatments for Metastatic Renal Cell Carcinoma: 10-Year Experience of Immunotherapy and Targeted Therapy. Cancer Res Treat.

[18] Moch H, Cubilla AL, Humphrey PA, Reuter VE, Ulbright TM (2016). The 2016 WHO Classification of Tumours of the Urinary System and Male Genital Organs-Part A: Renal, Penile, and Testicular Tumours. Eur Urol.

[19] Moch H, Artibani W, Delahunt B, Ficarra V, Knuechel R, Montorsi F, Patard JJ, Stief CG, Sulser T, Wild PJ (2009). Reassessing the current UICC/AJCC TNM staging for renal cell carcinoma. Eur Urol.

[20] Printz C (2010). New AJCC cancer staging manual reflects changes in cancer knowledge. Cancer.

[21] Eisenhauer EA, Therasse P, Bogaerts J, Schwartz LH, Sargent D, Ford R, Dancey J, Arbuck S, Gwyther S, Mooney M, Rubinstein L, Shankar L, Dodd L (2009). New response evaluation criteria in solid tumours: revised RECIST guideline (version 1.1). Eur J Cancer.

[22] Kim SH, Kim S, Joo J, Seo HK, Joung JY, Lee KH, Chung J (2016). A retrospective study of predictive factors for unexpectedly prolonged or shortened progression-free survival and overall survival among patients with metastatic renal cell carcinoma who received first-line targeted therapy. BMC Cancer.

[23] Tanaka N, Mizuno R, Yasumizu Y, Ito K, Shirotake S, Masunaga A, Ito Y, Miyazaki Y, Hagiwara M, Kanao K, Mikami S, Nakagawa K, Momma T (2017). Prognostic value of neutrophil-to-lymphocyte ratio in patients with metastatic renal cell carcinoma treated with first-line and subsequent second-line targeted therapy: A proposal of the modified-IMDC risk model. Urol Oncol.

[24] Kwon WA, Cho IC, Yu A, Nam BH, Joung JY, Seo HK, Lee KH, Chung J (2013). Validation of the MSKCC and Heng risk criteria models for predicting survival in patients with metastatic renal cell carcinoma treated with sunitinib. Ann Surg Oncol.

[25] Ko JJ, Xie W, Kroeger N, Lee JL, Rini BI, Knox JJ, Bjarnason GA, Srinivas S, Pal SK, Yuasa T, Smoragiewicz M, Donskov F, Kanesvaran R (2015). The International Metastatic Renal Cell Carcinoma Database Consortium model as a prognostic tool in patients with metastatic renal cell carcinoma previously treated with first-line targeted therapy: a population-based study. Lancet Oncol.

[26] Petrelli F, Coinu A, Vavassori I, Cabiddu M, Borgonovo K, Ghilardi M, Lonati V, Barni S (2016). Cytoreductive Nephrectomy in Metastatic Renal Cell Carcinoma Treated With Targeted Therapies: A Systematic Review With a Meta-Analysis. Clin Genitourin Cancer.

[27] Heng DY, Wells JC, Rini BI, Beuselinck B, Lee JL, Knox JJ, Bjarnason GA, Pal SK, Kollmannsberger CK, Yuasa T, Srinivas S, Donskov F, Bamias A (2014). Cytoreductive nephrectomy in patients with synchronous metastases from renal cell carcinoma: results from the International Metastatic Renal Cell Carcinoma Database Consortium. Eur Urol.

[28] Biswas B, Dabkara D, Ganguly S, Eswaran P, Ghosh J (2017). Cytoreductive Nephrectomy in Metastatic Renal Cell Carcinoma in the Era of Targeted Therapy: Scientifically Relevant or Natural Selection?. J Clin Oncol.

[29] Pindoria N, Raison N, Blecher G, Catterwell R, Dasgupta P (2017). Cytoreductive nephrectomy in the era of targeted therapies: a review. BJU Int.

