# Supplementary material for: A retrospective comparative study of progression-free survival and overall survival between metachronous and synchronous metastatic renal cell carcinoma in intermediate- or poor-risk patients treated with VEGF-targeted therapy
Source: Oncotarget. 2017 Sep 6;8(55):93633–43. doi: 10.18632/oncotarget.20674 (PMC5706824; doi:10.18632/oncotarget.20674)
Supplement: Supplementary file 1 [file oncotarget-08-93633-s001.pdf]

A retrospective comparative study of progression-free survival and overall survival between metachronous and synchronous metastatic renal cell carcinoma in intermediate- or poor-risk patients treated with VEGF-targeted therapy

SUPPLEMENTARY MATERIALS

Supplementary Table 1: Baseline characteristics table (N=118)

	N(%) or Median (min-max)
Age (years)	58 (34-81)
Gender (male/female)	94 (79.7)/24 (20.3)
Nephrectomy	54 (45.8)
Metastatectomy	23 (19.5)
Treatment free interval, ≥1yr/ <1yr	27 (22.9)/91 (77.1)
anemia	86 (72.9)
hypercalcemia	15 (12.7)
Neutrophilia	22 (18.6)
elevated LDH	26 (22.0)
KPS > 80 /≤ 80	1115 (97.5)/3 (2.5)
Thrombocytosis	14 (11.9)
Serum creatinine (mg/dL)	1.2 (0.6-3.8)
Estimated GFR (mL/min/1.73 m)	68.8 (22.3-142.1)
Metastatic lesion	
Lung	85 (72.0)
Liver	25 (21.2)
Lymph node	50 (42.4)
Bone	42 (35.6)
Brain	15 (12.7)
Number of metastatic lesions	2 (1-4)
Heng, Intermediate risk	93 (78.8)
Poor risk	25 (21.2)
CT or pT stage, T1 - T2/ T3 -T4	51 (43.2)/37 (31.4)
unknown	30 (25.4)
cN or pN stage, N0/ N1/ Nx	30 (25.4)/30 (25.4)/15 (12.7)
unknown	43 (36.4)
Synchronous / Metachronous mRCC	86 (72.9)/32 (27.1)
Fuhrman nuclear grade, G1-G2 / G3-G4	25 (21.2)/55 (46.6)
unknown	38 (32.2)
Treatment duration (Month)	5.0 (1.0-62.0)
Follow-up duration (Month)	59.3 (4.8-61.3)
Progression free survival (Month)	5.6 (1.0-62.0)
Overall survival (=Cancer specific survival, Month)	12.8 (1.0-87.2)
Survival/death	20 (17.0)/98 (83.0)
